# Skeletal Muscle Depletion Predicts the Prognosis of Patients with Advanced Pancreatic Cancer Undergoing Palliative Chemotherapy, Independent of Body Mass Index

**DOI:** 10.1371/journal.pone.0139749

**Published:** 2015-10-05

**Authors:** Younak Choi, Do-Youn Oh, Tae-Yong Kim, Kyung-Hun Lee, Sae-Won Han, Seock-Ah Im, Tae-You Kim, Yung-Jue Bang

**Affiliations:** 1 Department of Internal Medicine, Seoul National University Hospital, Seoul, Korea; 2 Cancer Research Institute, Seoul National University College of Medicine, Seoul, Korea; Centro Nacional de Investigaciones Oncológicas (CNIO), SPAIN

## Abstract

**Introduction:**

Body composition has emerged as a prognostic factor in cancer patients. We investigated whether sarcopenia at diagnosis and loss of skeletal muscle during palliative chemotherapy were associated with survival in patients with pancreatic cancer.

**Methods:**

We retrospectively reviewed the clinical outcomes of pancreatic cancer patients receiving palliative chemotherapy between 2003 and 2010. The cross-sectional area of skeletal muscle at L3 by computed tomography was analyzed with Rapidia 3D software. We defined sarcopenia as a skeletal muscle index (SMI)< 42.2 cm^2^/m^2^ (male) and < 33.9 cm^2^/m^2^ (female) using ROC curve.

**Results:**

Among 484 patients, 103 (21.3%) patients were sarcopenic at diagnosis. Decrease in SMI during chemotherapy was observed in 156 (60.9%) male and 65 (40.6%) female patients. Decrease in body mass index (BMI) was observed in 149 patients (37.3%), with no gender difference. By multivariate analysis, sarcopenia (*P*< 0.001), decreasedBMI and SMI during chemotherapy (*P* = 0.002, *P* = 0.004, respectively) were poor prognostic factors for overall survival (OS). While the OS of male patients was affected with sarcopenia (*P*< 0.001) and decreased SMI (*P* = 0.001), the OS of female patients was influenced with overweight at diagnosis (*P* = 0.006), decreased BMI (*P* = 0.032) and decreased SMI (*P* = 0.014). Particularly, while the change of BMI during chemotherapy did not have impact on OS within the patients with maintained SMI (*P* = 0.750), decrease in SMI was associated with poor OS within the patients with maintained BMI (HR 1.502; *P* = 0.002).

**Conclusions:**

Sarcopenia at diagnosis and depletion of skeletal muscle, independent of BMI change, during chemotherapy were poor prognostic factors in advanced pancreatic cancer.

## Introduction

The pancreas, which plays a major role in digestion, produces various digestive enzymes. Therefore, most patients with pancreatic cancer have a history of unintentional weight loss at presentation. Numerous reports over several decades have shown that weight loss in cancer patients predicts poor survival [1]. We recently reported that weight loss during palliative chemotherapy predicts poor survival in advanced pancreatic cancer (APC) patients [2]. However, recent studies have reported that the change in body composition rather than body weight is important for predicting all-cause mortality of patients with cancer [3–8].

Cachexia can be diagnosed in patients with more than 5% of involuntary loss of body weight over the previous 6 months or in already depleted patients (body-mass index [BMI] <20 kg/m^2^) or sarcopenic patients with more than 2% of ongoing weight loss [9]. Sarcopenia is defined as the state of depleted muscle mass independent of fat mass. The current diagnostic criterion for sarcopenia is muscle mass over 2 standard deviations (SD) below that for typical healthy adults (men, 7.26 kg/m^2^; women, 5.45 kg/m^2^; by dual-energy x-ray absorptiometry [DEXA] in the western population) [9, 10]. Loss of skeletal muscle and gain of adipose tissue can occur simultaneously, which results in sarcopenic obesity. Sarcopenic obesity was recently shown to be an independent poor prognostic factor for survival in patients with cancer [3–6]. However, sarcopenia defined by the cutoffs from previous Western studies could not show the relationship with survival among the Asian population [11]. Furthermore, no study has investigated the dynamics of body weight and body muscle mass in cancer patients who receive chemotherapy and its clinical implication.

In this study, we investigated the prevalence of sarcopeniaby the cutoffs from our ownpopulation and changes in skeletal muscle mass during palliative chemotherapy, and their impact on clinical outcomes in patients with APC.

## Materials and Methods

### Study patients

We assessed all the patients with histologically confirmed pancreatic cancer (PC) who received palliative chemotherapy at Seoul National University Hospital between January 2003 and December 2012. Among the assessed patients, those without computed tomography (CT) scans that included the third lumbar (L3) level within 30 days before starting the first cycle of chemotherapy were excluded.

The patients’ medical records were reviewed retrospectively. Body weight and height were collected at the time of diagnosis of APC. Change in body weight was determined asthe difference in the weight between atdiagnosis and at the cessation of first-line chemotherapy because of progression. Patients were categorized according to initial BMI into 3 groups, as follows: <20.0 kg/m^2^ (underweight), 20.0–24.9 kg/m^2^ (normal weight), and ≥25.0 kg/m^2^ (overweight or obese).

### Skeletal muscle mass measurement

**Fig 1:**
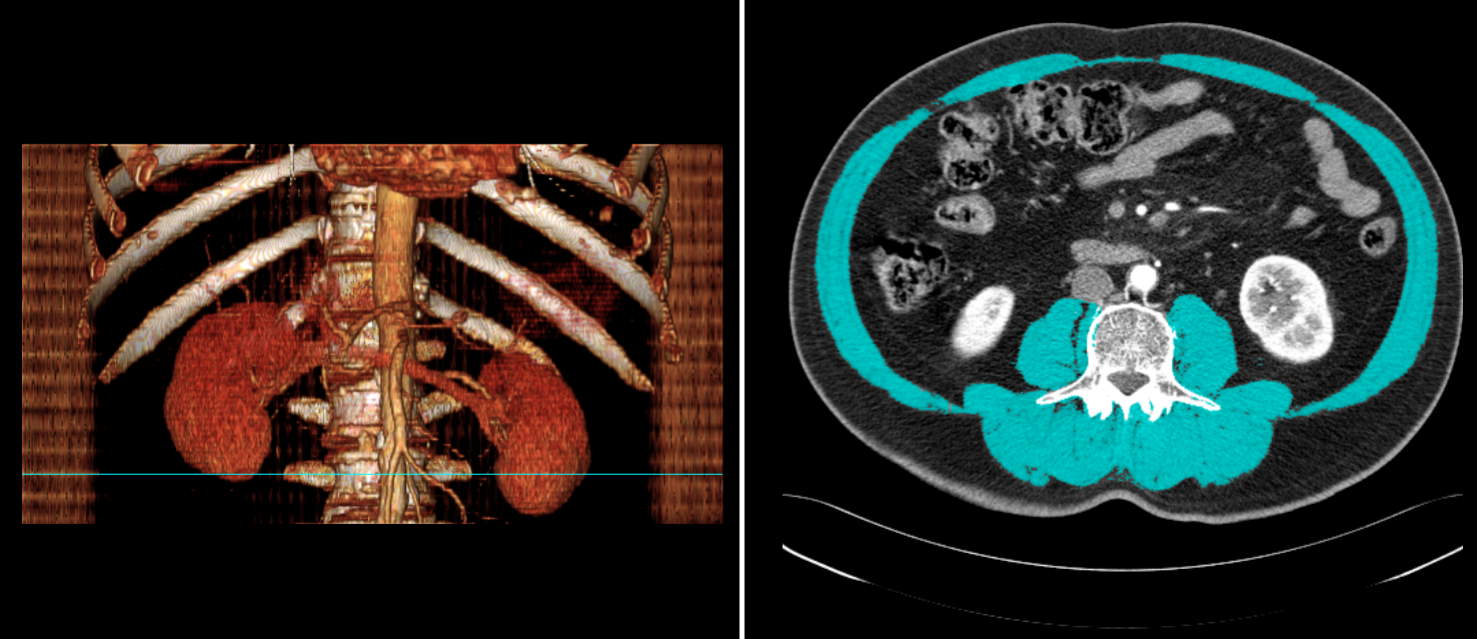
Computed tomography images of the region of the third lumbar vertebra, with skeletal muscle highlighted in blue (-29 to 150 Hounsfield units). The blue line on the three-dimensionally reconstructed image on the left indicates the level of the third lumbar vertebra, which is shown in the axial view on the right.

Cross-sectional imaging of CT scans was used to analyze skeletal muscle mass as in previous studies [3, 4, 12]. CT scans performed at the date of diagnosis wereused to quantify the initial area of L3 skeletal muscle. The CT scans that provided diagnostic evidence of disease progression were used for the final measurement. Two adjacent axial images within the same serieswere used to determine the cross-sectional area of L3 skeletal muscle, and the mean of the 2 measurements was calculated for each patient. Muscles were quantified within a Hounsfield unit (HU) range of -29 to 150 HU [12], and analyzed with Rapidia 3D software (v2.8; INFINITT Healthcare, Seoul, Korea). A single trained staff person corrected the boundary of the entire area of L3 skeletal muscle (Fig 1). Muscle area was normalized for height in meters squared (m^2^) and reported as the lumbar skeletal muscle index (SMI) (cm^2^/m^2^) [4, 5, 9]. We used the area under the receiver operating characteristic (ROC) curve to determine the cutoff values ofsarcopenia for both genders.

### Statistical analysis

Data on baseline characteristics and body composition are shown as mean and SD. The median overall survival (OS) and progression free survival (PFS) were determined using the Kaplan-Meier method. Between-group differences in demographic and clinical data were evaluated using the Fisher exact test for categorical variables and independent *t*-test for continuous variables. Relationships between continuous variables were assessed by determining Pearson correlations. Logistic regression was used to identify factors associated with decrease in SMI during chemotherapy. Comparison of survival among subgroups was done using the log-rank test. Cox proportional hazards regression model was used to determine the relationship of explanatory variables to survival as hazard ratios (HR) and 95% confidence intervals (CI). All tests were 2-sided, and *P*≤0.05 was considered statistically significant. Statistical analyses were performed using SPSS, ver. 19.0 (IBM Corp. Armonk, NY, USA).

#### Ethics

This study was reviewed and approved by the Institutional Review Board of Seoul National University Hospital (IRB No: H-1307-146-507). All studies were conducted according to ethical guidelines (Declaration of Helsinki) for biomedical research. Requirement for informed consent was waived for this retrospective analysis of clinical data. All patients’records were anonymized and de-identified prior to analysis.

## Results

### Patient characteristics

**Fig 2:**
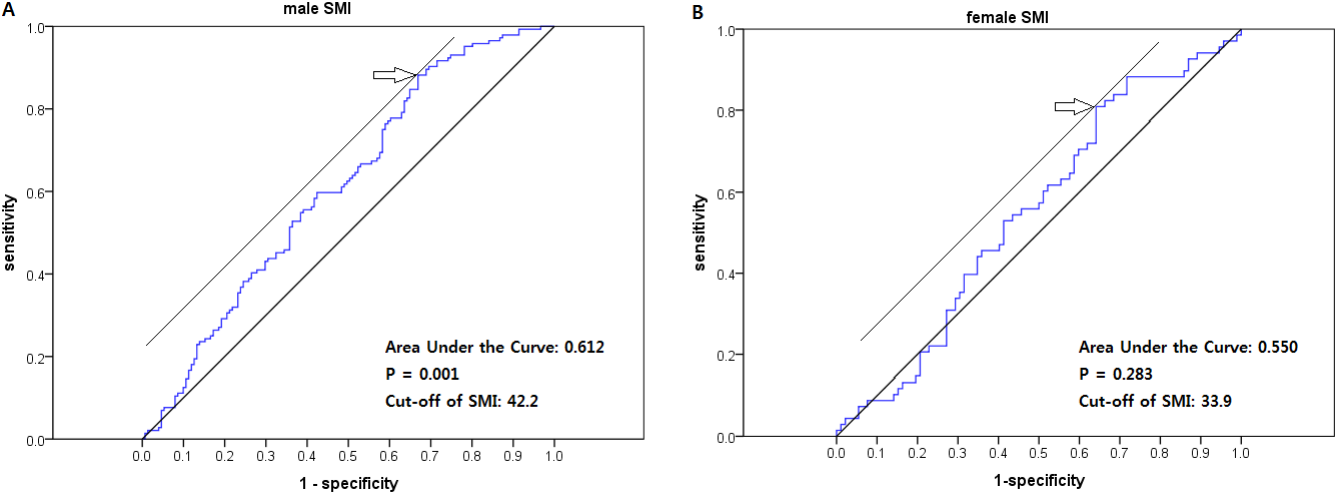
ROC curve ofsarcopenia for both genders.

Of 514 initially identified consecutive patients, this study included 484 patients with adequate CT images. Their clinical characteristics at initial assessment are shown according to gender in Table 1. The median age was 60.4 years (range, 20–85 years) and 80.2% of patients had metastatic disease. Most patients (90.7%) underwent gemcitabine-based chemotherapy, and the remainders received fluoropyrimidine-based chemotherapy including 8 patients treated with FOLFIRINOX regimen. The cutoff values of sarcopenia were determined through ROC curve were< 42.2 cm^2^/m^2^ for men and < 33.9 cm^2^/m^2^ for women, respectively (Fig 2).

**Table 1:** Clinical characteristics by gender.

Characteristic		Men(N = 295)		Women(N = 189)		Total(N = 484)		*P*
		N	%	N	%	N	%	
Age, year	Mean	60.1		60.7		60.4		0.495^b^
	SD	10.1		9.8		9.9		
Extent of disease	LAPC	49	16.6	47	24.9	96	19.8	0.026^a^
	MPC	246	83.4	142	75.1	388	80.2	
ECOGPS	0–1	240	81.4	153	81.0	393	81.2	0.912^a^
	≥2	55	18.6	36	19.0	91	18.8	
BMI at diagnosis, kg/m^2^(N = 480)	Mean	21.7		21.9		21.8		0.509^b^
	SD	2.5		2.8		2.7		
BMI at diagnosis (N = 480)	<20 kg/m^2^	70	23.9	48	25.7	118	24.6	0.527^a^
	20–24.9 kg/m^2^	192	65.5	114	61.0	306	63.8	
	≥25kg/m^2^	31	10.6	25	13.3	56	11.6	
SMI at diagnosis, cm^2^/m^2^	Mean	47.7		38.6		44.0		< 0.001^b^
	SD	7.1		5.4		7.8		
Sarcopenia†	Yes	67	22.7	36	19.0	103	21.3	0.364^a^
	No	228	77.3	153	81.0	381	78.7	

LAPC, locally advanced pancreatic cancer; MPC, metastatic pancreatic cancer; ECOGPS, Eastern Cooperative Oncology Group performance status;BMI, body mass index; SMI, skeletal muscle index; SD, standard deviation.

^†^Sarcopenia: males< 42.2 cm^2^/m^2^, females< 33.9 cm^2^/m^2^.

^*a*^ *P*values were calculated using theFisher’s exact test.

^*b*^ *P*values were calculated using theIndependent t-test.

### Body composition

There were no significant differences in the mean BMI values and distribution of patients in BMI subgroups based on genders. The number of overweight or obese (BMI≥25 kg/m^2^) men and women were 10.6% and 13.3%, respectively. The mean SMI values (±SD) of men and women were 47.7±7.1 cm^2^/m^2^ and 38.6±5.4 cm^2^/m^2^, respectively (*P*<0.001). However, there were no significant differences in the distribution of men and women in SMI subgroups categorized with means and SDs.

Sarcopenia was found in 103 (21.3%) patients. The distribution of sarcopeniawas not significantly different between men and women(22.7% vs19.0%; *P* = 0.364,Table 1). There were 3 (0.6%) patients with sarcopenic obesity (overweight or obese patients with sarcopenia) and 48 (10.0%) patients with obvious cachexia (BMI<20 kg/m^2^ with sarcopenia; S1 Table), including 18 female patients. SMI at diagnosis was significantly correlated with BMI at diagnosis in both genders (males, *r* = 0.619, *P*<0.001; females, *r* = 0.506, *P*<0.001; S1A Fig).

The mean BMI loss during chemotherapy for each gender was 0.6 kg/m^2^, and one-third of each gender lost more than 1 kg/m^2^ (<-1 kg/m^2^). The mean SMI loss was significantly greater in men than in women (3.8 cm^2^/m^2^ vs 1.1 cm^2^/m^2^; *P*<0.001). More male patients lost greater than 2cm^2^/m^2^ (<-2 cm^2^/m^2^) than female patients (60.9% vs 40.6%; *P*<0.001; Table 2).

**Table 2:** Changes in body composition during first line chemotherapy by gender.

Characteristic		Men (N = 295)		Women (N = 189)		Total (N = 484)		*P*
		N	%	N	%	N	%	
Change in BMI, kg/m^2^ (N = 400)	Mean	-0.6		-0.6		-0.6		0.753^b^
	SD	1.8		1.9		1.9		
Change in BMI† (N = 400)	Decreased	94	37.6	55	36.7	149	37.3	0.852^a^
	Maintained	156	62.4	95	63.3	251	62.7	
Change in SMI, cm^2^/m^2^(N = 416)	Mean	-3.8		-1.1		-2.8		< 0.001^b^
	SD	5.4		4.2		5.1		
Change in SMI‡ (N = 416)	Decreased	156	60.9	65	40.6	221	53.1	< 0.001^a^
	Maintained	100	39.1	95	59.4	195	46.9	

BMI, body mass index; SMI, skeletal muscle index; SD, standard deviation.

^†^Change in BMI: maintained ≥ -1 kg/m^2^, decreased < -1 kg/m^2^.

^‡^Change in SMI: maintained ≥ -2 cm^2^/m^2^; decreased < -2 cm^2^/m^2^.

^*a*^ *P* values were calculated using theFisher exact test.

^*b*^ *P*values were calculated using theIndependent t-test.

### Factors related to decrease in skeletal muscle

Logistic regression analysis revealed that male gender, sarcopenia, decreased BMI during chemotherapy, and progressed tumor response were significantly associated with decreased SMI during first-line chemotherapy (S2 Table).

### Treatment outcomes

The median duration of follow-up was 11.0 months (95% CI 10.1–11.9 months). The median OS and PFS of first line chemotherapy were 8.4 months (95% CI 7.6–9.2 months) and 3.7 months (95% CI 3.1–4.2 months), respectively. The objective response rate and disease control rate were 21.9% and 60.5%, respectively.

#### Factors related to overall survival

**Fig 3:**
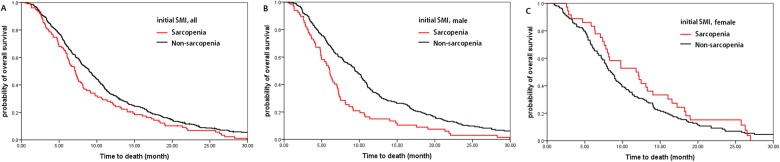
Survival according to skeletal muscle index. Initial SMI was related to worse prognosis (A) for the entire patients (HR 1.721; *P* < 0.001); (B) for the male patients (HR 1.736; *P* < 0.001); (C) not for female patients (*P* = 0.299).

**Fig 4:**
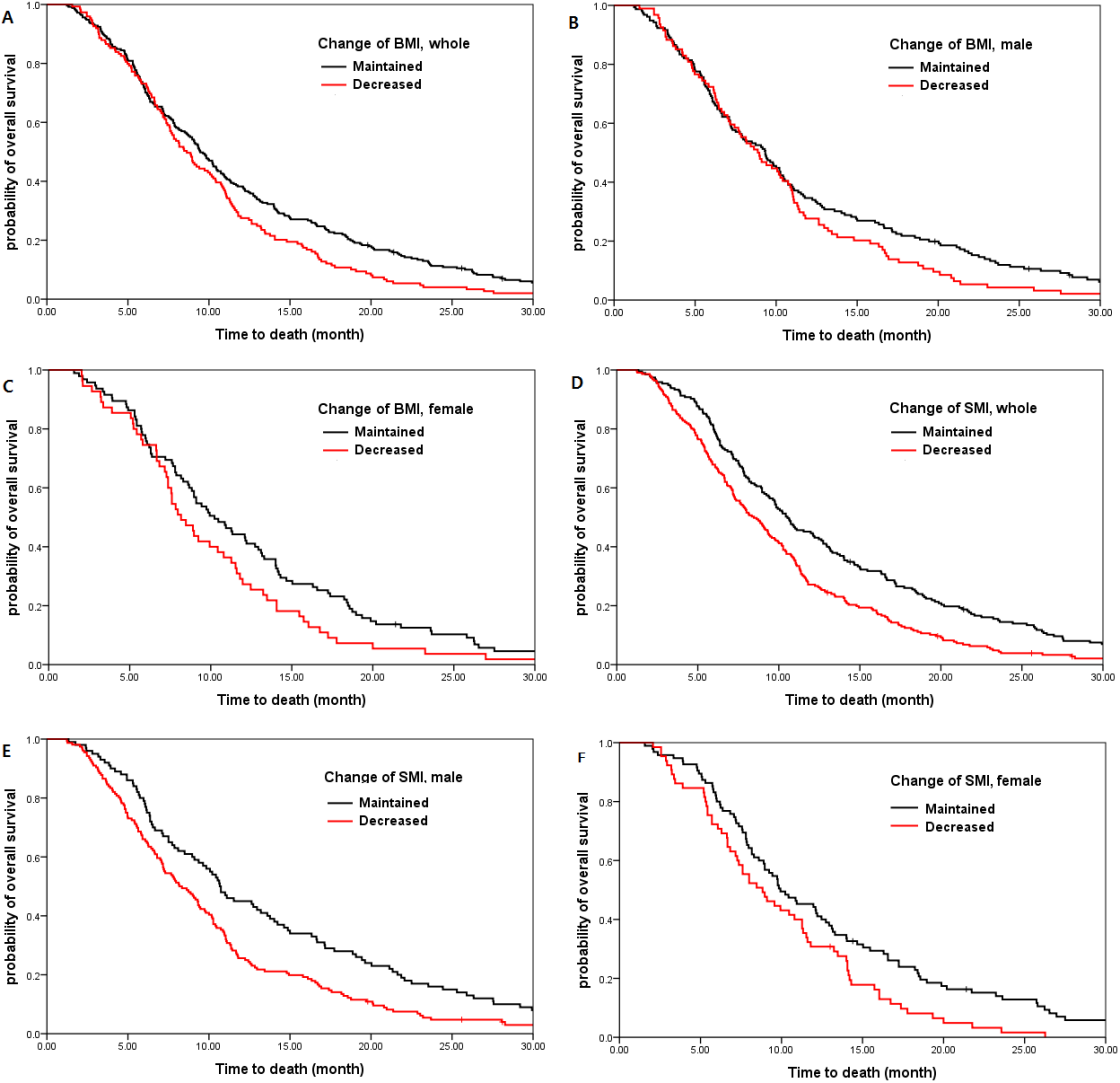
Survival according to change in body mass indexand skeletal muscle index. BMI decreased by more than 1 kg/m^2^ and SMI decreased by more than 2 cm^2^/m^2^ were strongly related to worseoutcome. Decrease of BMI (A) for the entire patients (HR 1.452; *P* = 0.002); (B) for the male patients (HR 1.218; *P* = 0.142); (C) for the female patients (HR 1.464; *P* = 0.032). Decrease of SMI (D) for the entire patients (HR 1.390;*P* = 0.004); (E) for the male patients (HR 1.560; *P* = 0.001); (F) for the female patients (HR 1.521; *P* = 0.014).

Table 3 shows the effect of multiple clinical factors on OS. By multivariate analysis, sarcopenia (HR 1.721; 95% CI 1.298–2.284; *P* < 0.001; Fig 3A), decreased BMI (HR 1.452; 95% CI 1.148–1.836; *P* = 0.002; Fig 4A), and decreased SMI (HR 1.390; 95% CI 1.109–1.742; *P* = 0.004; Fig 4D) were associated with poor survival. For male patients, sarcopenia (median OS of 6.1 vs 9.4 months; HR 1.736, 95% CI 1.310–2.300; *P* < 0.001; Fig 3B) and decreasedSMI (median OS of 8.2 vs 10.7 months; HR 1.560, 95% CI 1.199–2.030; *P* = 0.001; Fig 4E) was strongly related to worse prognosis. However,decreased BMI(median OS 8.9 vs 9.3 months; HR 1.218, 95% CI 0.936–1.585; *P* = 0.142; Fig 4B) showed a trend to worse prognosisand BMI at diagnosis did not influence on OS (*P* = 0.357). For female patients, both overweight at diagnosis (HR2.069, 95% CI 1.232–3.475; *P* = 0.006) and decreased BMI (median OS 8.2 vs 10.2 months; HR 1.464, 95% CI 1.034–2.071; *P* = 0.032; Fig 4C) were significantly associated with worse prognosis. However, while decreased SMI(medianOS 8.8 vs 9.9 months; HR 1.521, 95% CI 1.090–2.124; *P* = 0.014; Fig 4F) waspoor prognostic, sarcopenia at diagnosis had no relation with OS (*P* = 0.299; Fig 3C) (Table 4).

**Table 3:** Factors associated with overall survival.

Characteristics		No. of deaths/	OS, months	Univariate			Multivariate		
		No. of patients	(95% CI)	HR	95% CI	*P*	HR	95% CI	*P*
Gender	Female	183/189	8.8 (7.8–9.9)	0.957	0.795–1.153	0.645	1.002	0.805–1.246	0.988
	Male	289/295	8.1 (7.0–9.2)	1			1		
Age	≥60	251/260	8.0 (7.0–9.0)	1.076	0.898–1.289	0.429	1.247	1.009–1.541	0.041
	<60	221/224	9.0 (7.8–10.1)	1			1		
Extent of disease	MPC	380/388	7.7 (7.0–8.4)	1.558	1.239–1.959	<0.001	1.505	1.158–1.957	0.002
	LAPC	92/96	12.1 (10.4–13.8)	1			1		
PS	≥2	90/91	6.0 (4.9–7.1)	1.797	1.423–2.270	<0.001	1.382	1.042–1.834	0.025
	0–1	382/393	9.2 (8.3–10.1)	1			1		
BMI at diagnosis						0.204			0.670
	<20 kg/m^2^	116/118	8.0 (7.3–8.4)	1		reference	1		reference
	20–24.9 kg/m^2^	298/306	9.2 (8.2–10.3)	0.848	0.683–1.051	0.133	0.996	0.768–1.292	0.975
	≥25kg/m^2^	54/56	6.4 (5.0–7.9)	1.020	0.736–1.413	0.904	1.164	0.777–1.744	0.462
Sarcopenia	Yes	101/103	7.2 (6.2–8.1)	1.297	1.039–1.618	0.021	1.721	1.298–2.284	<0.001
	No	371/381	9.0 (8.1–9.9)	1			1		
Change in BMI	Decreased	148/149	8.6 (7.5–9.8)	1.266	1.030–1.555	0.025	1.452	1.148–1.836	0.002
	Maintained	247/251	9.5 (8.5–10.4)	1			1		
Change in SMI	Decreased	217/221	8.5 (7.3–9.6)	1.512	1.240–1.844	< 0.001	1.390	1.109–1.742	0.004
	Maintained	192/195	10.5 (9.1–12.0)	1			1		
Best response	Controlled†	283/293	11.7 (10.7–12.6)	0.256	0.210–0.313	< 0.001	0.265	0.206–0.340	< 0.001
	Progressed	189/191	4.9 (4.3–5.5)	1			1		

LAPC, locally advanced pancreatic cancer; MPC, metastatic pancreatic cancer; PS, performance status;BMI, body mass index; SMI, skeletal muscle index; OS, overall survival; HR, hazard ratio; CI, confidential interval.

^†^Controlled: complete response, partial response, and stable disease.

**Table 4:** Body compositionanalyses for predictors of overall survivalby gender groups.

Characteristics		Male				Female			
		OS, months	HR	95% CI	*P* ^a^	OS, months	HR	95% CI	*P* ^a^
BMI at diagnosis					0.357				0.005
	<20 kg/m^2^	7.2	1		reference	8.4	1		reference
	20–24.9 kg/m^2^	9.1	0.816	0.616–1.079	0.154	9.2	0.979	0.688–1.393	0.908
	≥25kg/m^2^	7.2	0.834	0.537–1.297	0.421	6.0	2.069	1.232–3.475	0.006
Change in BMI	Decreased	8.9	1.218	0.936–1.585	0.142	8.2	1.464	1.034–2.071	0.032
	Maintained	9.3	1			10.2	1		
Sarcopenia	Yes	6.1	1.736	1.310–2.300	< 0.001	11.8	0.818	0.559–1.196	0.299
	No	9.4	1			8.5	1		
Change in SMI	Decreased	8.2	1.560	1.199–2.030	0.001	8.8	1.521	1.090–2.124	0.014
	Maintained	10.7	1			9.9	1		

BMI, body mass index; SMI, skeletal muscle index; OS, overall survival; HR, hazard ratio; CI, confidential interval.

^*a*^ *P*values were calculated using the Cox-proportional hazard model, adjusted with age, extent of disease, and PS.

#### Correlation of BMI and SMI at diagnosis

Further analysis categorizing BMI at diagnosis into overweight or obese (≥25 kg/m^2^) or not (<25 kg/m^2^) and SMI at diagnosis into nonsarcopenia or sarcopenia revealed that median OS of sarcopenic male patients was reduced by 3.8 months against nonsarcopenic male patients eventhough they are not overweight or obse (median OS 6.0 months vs 9.8 months; HR1.746; *P*< 0.001). On the other hands, median OS of overweight or obese female patients was reduced by 3.0 months against not overweight female patients eventhough they are notinsarcopenic condition (median OS 6.0 months vs 9.0 months; HR 1.964; *P* = 0.003) (Table 5).

**Table 5:** Correlation of BMI and SMI at diagnosis.

			BMI at diagnosis			*P*
			<25kg/m^2^, N = 424	≥25kg/m^2^, N = 56		
SMI at diagnosis		Nonsarcopenia, N = 378	HR, 1; reference	HR, 1.320; *P* = 0.072^a^	(*P* = 0.054)^a^ ^,^ ^c^	0.013^a^
			OS = 9.4 months, N = 325	OS = 6.7 months, N = 53		
		Sarcopenia, N = 102	HR, 1.341; *P* = 0.012^a^	HR, 2.862; *P* = 0.073^a^	(*P* = 0.348)^a^ ^,^ ^d^	
			OS = 7.2 months, N = 99	OS = 6.1 months, N = 3		
SMI at diagnosis	Males (N = 293)	Nonsarcopenia, N = 226	HR, 1; reference	HR, 1.050; *P* = 0.817^b^	(*P* = 0.839)^b^ ^,^ ^c^	0.002^b^
			OS = 9.8 months, N = 197	OS = 7.2 months, N = 29		
		Sarcopenia, N = 67	HR, 1.746; *P* < 0.001^b^	HR, 2.250; *P* = 0.260^b^	(*P* = 0.816)^b^ ^,^ ^d^	
			OS = 6.0 months,N = 65	OS = 6.1 months, N = 2		
	Females (N = 187)	Nonsarcopenia, N = 152	HR, 1; reference	HR, 1.964; *P* = 0.004^b^	(*P* = 0.003)^b^ ^,^ ^c^	0.002^b^
			OS = 9.0 months, N = 128	OS = 6.0 months, N = 24		
		Sarcopenia, N = 35	HR, 0.861; *P* = 0.460^b^	HR, 11.719; *P* = 0.017^b^	(*P* = 0.013)^b^ ^,^ ^d^	
			OS = 12.1 months, N = 34	OS = 2.9 months, N = 1		

BMI, body mass index; SMI, skeletal muscle index; HR, hazard ratio; OS, overall survival.

^*a*^ *P*values were calculated using the Cox-proportional hazard model, adjusted with gender, age, extent of disease, and PS.

^*b*^ *P*values were calculated using the Cox-proportional hazard model, adjusted with age, extent of disease, and PS.

^*c*^ *P*values were calculated within nonsarcopenia group.

^*d*^ *P*values were calculated within sarcopenia group.

#### Correlation of changes in BMI and SMI

Although decreased BMI was an important factor associated with decrease in SMI (HR 2.520; *P*<0.001; S2 Table), the distribution of change in BMI was not well correlated with the distribution of change in SMI in either men (*r* = 0.165) or women (*r* = 0.347; S1B Fig).

Analysis of patients in SMI and BMI subgroups (SMI and BMI both maintained during chemotherapy, SMI maintained and BMI decreased, SMI decreased and BMI maintained, SMI and BMI both decreased) revealed that the patients with decreased SMI had shorter survival regardless of changes in BMI(*P* = 0.002; Table 6).

**Table 6:** Correlation of changesin BMI and SMI.

			BMI			*P*
			Maintained (N = 273)	Decreased (N = 141)		
SMI		Maintained, N = 195	HR, 1; reference	HR, 1.051; *P* = 0.789^a^	(*P* = 0.750)^a^ ^,^ ^c^	< 0.001^a^
			OS = 10.7 months, N = 156	OS = 9.2 months, N = 39		
		Decreased, N = 219	HR, 1.502; *P* = 0.002^a^	HR, 1.787; *P* < 0.001^a^	(*P* = 0.290)^a^ ^,^ ^d^	
			OS = 8.0 months, N = 117	OS = 8.6 months, N = 102		
SMI	Males (N = 254)	Maintained, N = 100	HR, 1; reference	HR, 1.029; *P* = 0.915^b^	(*P* = 0.744)^b^ ^,^ ^c^	0.005^b^
			OS = 10.7 months, N = 81	OS = 10.1 months, N = 19		
		Decreased, N = 154	HR, 1.586; *P* = 0.004^b^	HR, 1.686; *P* = 0.003^b^	(*P* = 0.544)^b^ ^,^ ^d^	
			OS = 7.8 months, N = 84	OS = 8.9 months, N = 70		
	Females (N = 160)	Maintained, N = 95	HR, 1; reference	HR, 1.023; *P* = 0.932^b^	(*P* = 0.650)^b^ ^,^ ^c^	0.065^b^
			OS = 10.9 months, N = 75	OS = 8.9 months, N = 20		
		Decreased, N = 65	HR, 1.267; *P* = 0.289^b^	HR, 1.849; *P* = 0.010^b^	(*P* = 0.180)^b^ ^,^ ^d^	
			OS = 9.1 months, N = 33	OS = 7.6 months, N = 32		

BMI, body mass index; SMI, skeletal muscle index; HR, hazard ratio; OS, overall survival

^*a*^ *P*values were calculated using the Cox-proportional hazard model, adjusted with gender, age, extent of disease, PS, BMI at diagnosis, and sarcopenia.

^*b*^ *P*values were calculated using the Cox-proportional hazard model, adjusted withage, extent of disease, PS, BMI at diagnosis, and sarcopenia.

^*c*^ *P*values were calculated within maintained SMI group.

^*d*^ *P*values were calculated within decreased SMI group.

Compared with a median OS of 10.7 months for male patients with both SMI and BMI maintained during chemotherapy, a decreased SMI was a poor prognostic indicator although BMI was maintained (median OS 7.8 months, HR 1.589; *P* = 0.004). For female patients,cachectic patients(BMI and SMI both decreased)showedthe worst OS (median OS 7.6 months, HR 1.849; *P* = 0.010). For the patients with maintained SMI, decreased BMI had no effect on survival (*P* = 0.750). Analyses according to each gender showed similar results.

## Discussion

In this study, we found that sarcopenia at diagnosis and skeletal muscle depletion during chemotherapy predicted worse survival for advanced pancreatic cancer patients.

The influence of weight at diagnosis and change of weight (BMI) during chemotherapy is still under debate issue. In our study, the meaning of BMI was different based on gender; forfemale patients, a BMI at diagnosis ≥25 kg/m^2^ conferred poor prognostic significance (HR 2.069; *P* = 0.006), and decreased BMI during chemotherapy had significantly worse effect on the survival in female patients (median OS 8.2 vs 10.2 months; *P* = 0.032), not male patients (Table 4).

Sarcopenia at diagnosis was observed in one-fifth of patients (22.7% of male patients and 19.0% of female patients; Table 1). The association of sarcopeniawith survival was particularly prominent in male patients (Fig 4; Table 4). Sarcopenic obesity, usually focused on Western studies, was worse than obesity without sarcopenia and did not result in better outcomes than cachexia [4–6]. In our study, there was a very few sarcopenic obese patients (0.6%), mainly because the prevalence of obesity is lower in Asian populations than in Western populations. We showed that the decreased SMI during chemotherapy was associated with poor survival (*P* = 0.004; Table 3) and cachectic change in body composition resulted in the worst outcome (*P*<0.001; Table 6). However, the most important finding of our study was that sarcopenic male patients at diagnosis had significantly shorter survival, independently of initial BMI (HR 1.746; *P*<0.001) and overweight or obese female patients at diagnosis had significantly shorter survival, independently of initial SMI (HR 1.964; *P* = 0.003; Table 5). Furthermore, while the change of BMI during chemotherapy did not have the statistically significant meaning in survival within the patients of maintained SMI (*P* = 0.750), decrease of SMI was associated with reduced survival within the patients of maintained BMI (HR 1.502; *P* = 0.002; Table 6). Several reports showed that not only cross-sectional area but also radiation attenuation value of muscle has potential prognostic meaning [5].

Inflammation, inactivity, low-protein intake and age-related factors are suggested as causes of sarcopenia. However, the mechanisms by which sarcopenia increases the risk of mortality remain unclear. Recently, in addition to the fact that glutamine is released from muscle and involved in production of rapid dividing immune cells [13], many signaling molecules, called as myokines, such as IL-6, IL-8, IL-15, brain-derived neurotrophic factor, leukemia inhibitory factor, follistatin-like 1, fibroblast growth factor-21, irisin, etc. have been identified to be produced by contracting myofibers, mediating the metabolic and endocrine function [14, 15]. Skeletal muscle is the largest organ accounting 40–50% of body weight and has high metabolic capacity using 20% of resting energy expenditure [16]. However, in the condition of muscle atrophy, energy expenditure is decreased and the rate of fat deposition tends to increase [17]. Reduced muscle mass and contractile dysfunction causes aberrant energy homeostasis, impaired cell growth, insulin resistance, and immune dysfunction by means of inadequate myokines and impaired amino acid metabolismin patients with sarcopenicobesty [15, 18, 19].

Clinically, increased susceptibility to infection, musculoskeletal injuries and other morbidities are known as a possible contributor to the shortened survival of sarcopenic patients [15, 20, 21]. Another obvious disadvantage of sarcopenia is the premature termination of treatment due to increased toxicities of chemotherapy. Because the dosage of chemotherapeutic agents is conventionally determined by body surface area or body weight, patients with sarcopenia masked by obesity, who have a lean body mass comparable to underweight patients, are prone to manifest chemotherapy toxicities [22–25]. Considering the vicious cycle of sarcopenia and increased morbidity and mortality, timely interventions to increase physical activity and muscle loading would be helpful for survival of cancer patients [15].

The main limitations of our study were the retrospective design and the threshold values used to identify sarcopenia. The most frequently used cutoffs of sarcopenia in the western population are 7.26 kg/m^2^ in men and 5.45 kg/m^2^ in women, which were obtained from the Gallagher et al’s study, which had beenconducted for young healthy adults aged 18–40 years by dual-energy x-ray absorptiometry (DEXA)[10, 26]. These were converted to CT measurements of 52.4 cm^2^/m^2^ for men and 38.5 cm^2^/m^2^ for women by a regression equationto correlate the area of L3 skeletal muscle as follows: appendicular skeletal muscle mass/height^2^ (kg/m^2^) = 0.11 × CT-derived SMI (cm^2^/m^2^) + 1.17; *r* = 0.89; *P*< 0.001) [3]. Sarcopeniadefined by these cutoffs were proved the potential as a powerful predictor of poor prognosis in Western studies [4–6]. However, we did not apply these values because recent Asian studies reported that the cutoffs of sarcopenia from the previous Western studieswere inappropriate for Asian patients [11, 27]. Furthermore, the mean SMIs of our study population were lower than those of Western cancer populations [4, 5]. At first, we have tried the cutoffs froma previous study of healthy young Korean adults of which age distribution was similar to Gallagher et al.’s study andcomposed of 1,245 men and 1,268 women [28]. In that study, thesex-specific cutoff values defining sarcopeniahave beensuggested as 6.58 kg/m^2^ for men and 4.59 kg/m^2^ for women, by DEXA, which was identified as <49.2cm^2^/m^2^ for men and <31.1cm^2^/m^2^ forwomen with CT scan by regression equations. However, these values allowed as many as 59.0% of male patients and only 6.9% of female patients to be sarcopenic state and these cutoffs could not obtain general consensus yet. To the best of our knowledge, there are no published cutoffs for sarcopeniain Asians. Therefore, we used the thresholds obtained from our own population by ROC curve, convincing that our study contains sufficiently large Asian population to try finding Asian cutoffs of sarcopenia. In our study, we obtained new cutoffs of < 42.2 cm^2^/m^2^ for men and < 33.9 cm^2^/m^2^ for women categorizing about one-fifth of both sex as sarcopenia. Although our study did not contain enough patients with sarcopenic obesity to perform statistically valid impact of sarcopenic obesity on survival, we could demonstrate the independent impact of SMI and BMI on survival. Our cutoffs for sarcopenia need to be valid through the additional analyseswith other Asian cancer population containing more sarcopenic obese patients.

## Conclusions

Sarcopenia at the diagnosis and the change in body composition during chemotherapy were independent predictors of poor survival in APC patients. Skeletal muscle depletion during chemotherapy was especially associated with poor prognosis, regardless of change in BMI. Assessment of body composition should be considered when treating patients with advanced pancreatic cancer.

## Supporting Information

S1 Fig Scatter plot for L3 lumbar skeletal muscle index and body mass index. (A) Relationship between L3 lumbar skeletal muscle index and body mass index (Pearson *r* = 0.455, *P* < 0.001 for all the patients; *r* = 0.619, *P* < 0.001 for men; *r* = 0.506, *P* < 0.001 for women). (B) Relationship between the changes in L3 lumbar skeletal muscle index and body mass index (*r* = 0.214, *P* <0.001 for all the patients; *r* = 0.165, *P* = 0.012 for men; *r* = 0.347, *P* < 0.001 for women).
(TIF)

S1 Table Prevalence of sarcopenia for each body mass index subgroup. (DOCX)

S2 Table Risk factors for decrease in skeletal muscle index. (DOCX)
