# Supplementary figures and images for: Skeletal Muscle Depletion Predicts the Prognosis of Patients with Advanced Pancreatic Cancer Undergoing Palliative Chemotherapy, Independent of Body Mass Index
Source: PLoS One. 2015 Oct 5;10(10):e0139749. doi: 10.1371/journal.pone.0139749 (PMC4593598; doi:10.1371/journal.pone.0139749)

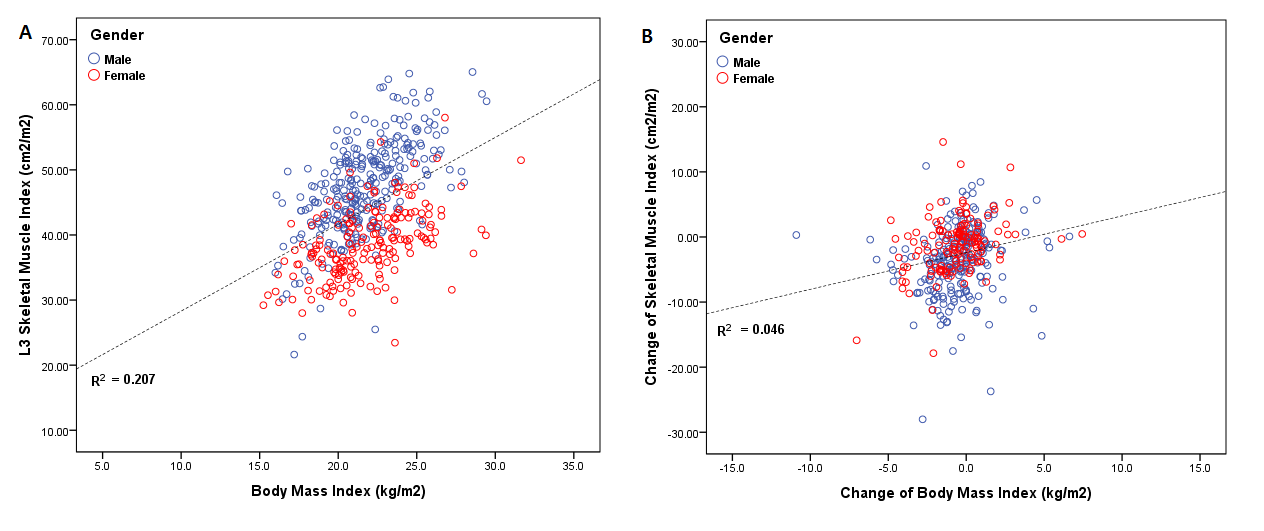

Supplement: S1 Fig — (A) Relationship between L3 lumbar skeletal muscle index and body mass index (Pearson r = 0.455, P < 0.001 for all the patients; r = 0.619, P < 0.001 for men; r = 0.506, P < 0.001 for women). (B) Relationship between the changes in L3 lumbar skeletal muscle index and body mass index (r = 0.214, P <0.001 for all the patients; r = 0.165, P = 0.012 for men; r = 0.347, P < 0.001 for women). (TIF) [file pone.0139749.s001.tif]
