# Supplementary material for: Skeletal Muscle Depletion Predicts the Prognosis of Patients with Advanced Pancreatic Cancer Undergoing Palliative Chemotherapy, Independent of Body Mass Index
Source: PLoS One. 2015 Oct 5;10(10):e0139749. doi: 10.1371/journal.pone.0139749 (PMC4593598; doi:10.1371/journal.pone.0139749)
Supplement: S1 Table — (DOCX) [file pone.0139749.s002.docx]

**S1 Table. Prevalence of sarcopenia for each body mass index subgroup**

| Characteristic | | BMI ≥ 25 kg/m^2^ (N = 56) | | BMI 20-24.9 kg/m^2^ (N = 306) | | BMI < 20 kg/m^2^ (N = 118) | | Total  (N = 480) | *P^a^* |
| --- | --- | --- | --- | --- | --- | --- | --- | --- | --- |
|  |  | Non-sarcopenia | Sarcopenia | Non-sarcopenia | Sarcopenia | Non-sarcopenia | Sarcopenia |  |  |
| Age | ≥ 60 | 32 (91.4%) | 3 (8.6%) | 125 (80.6%) | 30 (19.4%) | 43 (63.2%) | 25 (36.8%) | 258 | 0.148 |
|  | < 60 | 21 (100%) | 0 | 130 (86.1%) | 21 (13.9%) | 27 (54.0%) | 23 (46.0%) | 222 |  |
| Gender | Male | 29 (93.5%) | 2 (6.5%) | 158 (82.3%) | 34 (17.7%) | 39 (55.7%) | 31 (44.3%) | 293 | 0.748 |
|  | Female | 24 (96.0%) | 1 (4.0%) | 97 (85.1%) | 17 (14.9%) | 31 (64.6%) | 17 (35.4%) | 187 |  |
| Total |  | 53 (94.6%) | 3 (5.4%) | 255 (83.3%) | 51 (16.7%) | 70 (59.3%) | 48 (40.7%) | 480 |  |

BMI, body mass index.

*^a^ P* values were calculated using the Fisher exact test.
