# Supplementary material for: Skeletal Muscle Depletion Predicts the Prognosis of Patients with Advanced Pancreatic Cancer Undergoing Palliative Chemotherapy, Independent of Body Mass Index
Source: PLoS One. 2015 Oct 5;10(10):e0139749. doi: 10.1371/journal.pone.0139749 (PMC4593598; doi:10.1371/journal.pone.0139749)
Supplement: S2 Table — (DOCX) [file pone.0139749.s003.docx]

**S2 Table. Risk factors for decrease† in skeletal muscle index**

|  |  | HR | 95% CI | *P^a^* |
| --- | --- | --- | --- | --- |
| Gender | Male | 2.374 | 1.536-3.668 | < 0.001 |
| Age | ≥ 60 | 0.776 | 0.505-1.193 | 0.247 |
| Extent of disease | MPC | 0.824 | 0.476-1.426 | 0.489 |
| ECOG PS | ≥ 2 | 1.475 | 0.808-2.693 | 0.205 |
| BMI at diagnosis |  |  |  | 0.560 |
|  | < 20 kg/m^2^ | 1 |  | reference |
|  | 20-24.9 kg/m^2^ | 0.851 | 0.506-1.429 | 0.541 |
|  | ≥ 25 kg/m^2^ | 0.663 | 0.313-1.403 | 0.282 |
| Sarcopenia† | Yes | 1.678 | 1.035-2.721 | 0.036 |
| Change in BMI‡ | Decreased | 2.520 | 1.515-4.192 | < 0.001 |
| Best response | Progressed | 2.209 | 1.356-3.600 | 0.001 |

MPC, metastatic pancreatic cancer; ECOG PS, Eastern Cooperative Oncology Group performance status; BMI, body mass index; HR, hazard ratio; CI, confidential interval.

† Sarcopenia: males < 42.2 cm^2^/m^2^, females < 33.9 cm^2^/m^2^.

‡ Change in BMI: decreased < -1 kg/m^2^.

*^a^ P* values were calculated using the Logistic regression.
